# Differential Gene Expression Profiling of Dystrophic Dog Muscle after MuStem Cell Transplantation

**DOI:** 10.1371/journal.pone.0123336

**Published:** 2015-05-08

**Authors:** Florence Robriquet, Aurélie Lardenois, Candice Babarit, Thibaut Larcher, Laurence Dubreil, Isabelle Leroux, Céline Zuber, Mireille Ledevin, Jack-Yves Deschamps, Yves Fromes, Yan Cherel, Laetitia Guevel, Karl Rouger

**Affiliations:** 1 INRA, UMR703 PAnTher, Nantes, France; 2 LUNAM Université, Oniris, École nationale vétérinaire, agro-alimentaire et de l’alimentation Nantes-Atlantique, Nantes, France; 3 Université de Nantes, Nantes, France; 4 Laboratoire RMN AIM-CEA, Institut de Myologie, Hôpital Pitié-Salpêtrière, Paris, France; University of Minnesota Medical School, UNITED STATES

## Abstract

**Background:**

Several adult stem cell populations exhibit myogenic regenerative potential, thus representing attractive candidates for therapeutic approaches of neuromuscular diseases such as Duchenne Muscular Dystrophy (DMD). We have recently shown that systemic delivery of MuStem cells, skeletal muscle-resident stem cells isolated in healthy dog, generates the remodelling of muscle tissue and gives rise to striking clinical benefits in Golden Retriever Muscular Dystrophy (GRMD) dog. This global effect, which is observed in the clinically relevant DMD animal model, leads us to question here the molecular pathways that are impacted by MuStem cell transplantation. To address this issue, we compare the global gene expression profile between healthy, GRMD and MuStem cell treated GRMD dog muscle, four months after allogenic MuStem cell transplantation.

**Results:**

In the dystrophic context of the GRMD dog, disease-related deregulation is observed in the case of 282 genes related to various processes such as inflammatory response, regeneration, calcium ion binding, extracellular matrix organization, metabolism and apoptosis regulation. Importantly, we reveal the impact of MuStem cell transplantation on several molecular and cellular pathways based on a selection of 31 genes displaying signals specifically modulated by the treatment. Concomitant with a diffuse dystrophin expression, a histological remodelling and a stabilization of GRMD dog clinical status, we show that cell delivery is associated with an up-regulation of genes reflecting a sustained enhancement of muscle regeneration. We also identify a decreased mRNA expression of a set of genes having metabolic functions associated with lipid homeostasis and energy. Interestingly, ubiquitin-mediated protein degradation is highly enhanced in GRMD dog muscle after systemic delivery of MuStem cells.

**Conclusions:**

Overall, our results provide the first high-throughput characterization of GRMD dog muscle and throw new light on the complex molecular/cellular effects associated with muscle repair and the clinical efficacy of MuStem cell-based therapy.

## Introduction

Duchenne Muscular Dystrophy (DMD) is a progressive and fatal X-linked recessive disorder of skeletal and cardiac muscles. It is the most common muscular dystrophy, affecting one in 3,500 male births [1], being characterized by a lack of dystrophin at the muscle fibre membrane [2,3]. Dystrophin is the essential link between the subsarcolemmal cytoskeleton and the extracellular matrix [4,5]. In dystrophin-deficient muscle, this connection is weakened and results in the partial disruption of sarcolemma exposed to high tension and an influx of extracellular Ca^2+^ that activates proteases. This leads to successive necrosis/regeneration cycles of the muscle fibres, so that, ultimately, necrotic fibres fail to regenerate and the muscle tissue is progressively replaced by connective and adipose tissue. Numerous observations suggest that the impaired structural role of the dystrophin-glycoprotein complex alone may be insufficient to account for the massive degenerative process and that the deregulation of intracellular signalling pathways concomitant with skeletal muscle tissue remodelling also participate in DMD pathogenesis [6–8].

Based on large-scale gene expression analysis, previous studies have described transcriptional changes in the skeletal muscle of individuals with DMD. The first study revealed an over-expression of developmentally regulated genes such as α-cardiac actin and a down-regulation of nuclear-encoded mitochondrial gene expression [9]. Another study suggested an up-regulation of insulin-like growth factors and the induction of ‘cardiac-associated’ genes in dystrophin-deficient muscle due to factors implicated in macrophage infiltration, activation of muscle satellite cells and non-regeneration-linked changes in myofibre homeostasis [10]. It is also well known that immune response signals and extracellular matrix genes, as well as genes encoding muscle structure and regeneration processes, are over-expressed in DMD muscle, thus reflecting the regenerative nature of the disease [11,12]. In a more recent study, Pescatori et al. (2007) described the gene expression signature that characterizes DMD muscle during the initial or presymptomatic phase of the disease, and showed the altered expression of genes involved in the inflammatory response, remodelling of the extracellular matrix, muscle regeneration and energy metabolism. Similar studies have been performed on the *mdx* mouse, a genetic and biochemical model of the human disease [13–16], confirming the presence of many alterations in the dystrophic gene expression profile. Marotta et al. (2009) showed a strong up-regulation of inflammation-related genes as well as genes related to cell adhesion, muscle structure/regeneration and extracellular matrix remodeling during *mdx* disease evolution [17]. More recently, a transcriptomic study has been performed using a dystrophic dog model, CXMD_J_, to analyse dystrophic diaphragm tissue in the neonatal period [18]. Microarrays have also been described as a useful tool to assess the effects of therapeutic approaches such as the antisense treatment, or the overexpression of utrophin in *mdx* mouse [19,20]. To investigate potential therapies for individuals with DMD, preclinical studies are commonly performed using the Golden Retriever Muscular Dystrophy (GRMD) dog model, which is characterized by rapid progressive clinical dysfunction and severe muscle tissue remodelling [21,22]. GRMD dogs present typical histological features of a dystrophic process with repeated cycles of muscle necrosis and regeneration, variability in fibre size, splitting and fibrosis [23]. Moreover, GRMD dogs show most of the signs found in the human disease, among them muscle wasting, postural abnormalities and premature death, allowing to consider GRMD dog as a relevant animal model for DMD [22–25]. Recently, proteomic profiling performed on GRMD dogs have identified signalling proteins implicated in secondary changes taking place in dystrophic muscles [6,26]. A quantitative proteomic analysis, performed on both cytoplasmic and phospho-enriched fractions, has identified 84 proteins as being differentially represented in GRMD *versus* healthy dog muscle implicated in muscle development and contraction, glycolytic and oxidative metabolism, calcium ion homeostasis, intracellular signalling and regulation of apoptosis [6]. Nevertheless, little is known at present about transcriptional alterations affecting molecular pathways in GRMD dogs. Indeed, only a single transcriptomic study has been carried out on GRMD dogs aiming to define the molecular signals that drive muscle hypertrophy in *Sartorius cranialis* muscle and the differential muscle involvement in the GRMD model [27]. Recently, we have shown that systemic delivery of MuStem cells, isolated using a modification of the procedure that led to the identification of Muscle-Derived Stem Cells [28], could represent an attractive avenue for future therapeutic applications in individuals with DMD. Indeed, when intra-arterially delivered in immunosuppressed GRMD dog, allogeneic MuStem cells contributed to muscle damage course limitation with an increased regeneration activity and an interstitial expansion restriction. Importantly, they allow persisting stabilization of dog’s clinical status defined by a clinical score maintained up to 70% of that of the healthy dogs more than 6 months after the cell transplantation, reflecting a poor fatigability, a low intensity of limb stiffness and ankylosis [29]. These effects are linked to a relative low dystrophin protein level and a low percentage of dystrophin-positive muscle fibres that clearly evoke the implication of other molecular mechanisms to explain the mode of action of the MuStem cells. Since expression profiling allows the simultaneous monitoring of a large number of biological processes, in the present study we used an undedicated transcriptional approach with gene-expression microarrays to analyse muscle samples from healthy, mock GRMD and MuStem cell-treated GRMD dogs (GRMD^MuStem^). This strategy is reinforced by the fact that such an approach can detect changes that are not histologically visible. We provide the first high-throughput characterization of the GRMD dog model and report insights into the molecular/cellular impact of MuStem cell delivery in a dystrophic context. Overall, we show that one remarkable outcome of MuStem cell delivery concerns the up-regulation of genes implicated in regeneration of muscle fibre correlated with histological remodelling. In addition, we find that these cells can act on several other biological pathways implicated in protein degradation mechanisms and energy metabolism.

## Materials and Methods

### Animals

This study was approved by the Ethics Committee on Animal Experimentation of the Pays de la Loire Region, France, in accordance with the guidelines from the French National Research Council for the Care and Use of Laboratory Animals (Permit Numbers: CEEA.2012.104) (S1 Cheklist). Pain evaluation was performed every day during a complete clinical evaluation by a veterinarian and an analgesia treatment was set up if necessary. Seventeen golden retrievers were included in the study. All the dogs were obtained from the Centre d'Elevage du Domaine des Souches (CEDS, Mézilles, France) and housed at the Boisbonne Centre for Gene and Cell Therapy of Oniris (Nantes, France). The dogs were housed in a controlled environment (temperature 21 ± 1°C, 12-h light/dark cycle). GRMD dogs display a single base change in the acceptor splice site of intron 6 of the dystrophin gene. Skipping of exon 7 disrupts the mRNA reading frame and results in premature termination of translation [30]. Affected dogs were identified in the first week of life using polymerase chain reaction (PCR)-based genotyping. This identification was confirmed by a dramatic and early rise in levels of serum creatine kinase [31]. Seven 10-week-old healthy dogs were dedicated to MuStem cell isolation. The 10 other dogs were used in the *in vivo* experiments at 9-month-old (3 healthy, 3 GRMD and 4 GRMD^MuStem^ dogs) as described in Table 1. Among the 10 dogs, two dogs (#6G and #9G^Mu^) died before the end of the protocol at 38 weeks and displayed severe lesions of aspiration pneumonia after necropsy examination. Therefore, these animals are excluded from the transcriptomic analysis because of their altered RNA profiles. One GRMD^MuStem^ dog (10G^Mu^) was maintained alive for long-term follow-up. Dogs were euthanized by intravenous administration of sodium pentobarbital (2000 mg, Dolethal, Vetoquinol SA, Magny Vernois).

**Table 1:** Description of the ten dogs used in the *in vivo* study.

Group	Dog identification	Necropsy age (weeks)	Immunosuppression	MuStem cell transplantation	Clinical scoring & Histopathological analyses	Microarray	RT-qPCR
**Healthy**	1H	38	None	None	X	X	X
	2H	41	None	None	X	X	X
	3H	41	None	None	X		X
**GRMD**	4G	41	CSA-AZA	None	X	X	X
	5G	40	CSA-AZA	None	X	X	X
	6G	38*	CSA-AZA	None	X		
**GRMD** ^**MuStem**^	7G^Mu^	40	CSA-AZA	X	X	X	X
	8G^Mu^	45	CSA-AZA	X	X	X	X
	9G^Mu^	38*	CSA-AZA	X	X		
	10G^Mu^	Still alive	CSA-AZA	X	X		

The table presents a description of the Healthy, GRMD and GRMD^MuStem^ dogs and gives details of the experiments carried out on each of the 10 dogs. "*" signifies that the considered dog died of aspiration pneumonia before the end of the protocol. "X" indicates that the considered experiment was performed.

### Canine MuStem cell isolation

Primary wild type MuStem cells, corresponding to delayed adherent stem cells, were isolated from a pool of hind limb muscles of 10-week-old dogs (n = 7; independent experiment) based on a preplating technique, as previously established [29]. Cells were incubated at 37°C with 5% CO_2_, maintained at roughly 75% of confluence to avoid spontaneous myogenic differentiation and passaged every 4 to 5 days. The medium was replaced every two days to allow for their expansion. MuStem cells correspond to early myogenic progenitors and uncommitted cells characterized by a large expansion capacity and an ability to differentiate into myogenic, osteogenic and adipogenic cells after *in vitro* cell lineage-specific induction.

### Immunosuppression of GRMD dogs

The mock GRMD (GRMD) and cell transplanted (GRMD^MuStem^) dogs were immunosuppressed with 27 mg/kg of oral cyclosporine (Neoral; Novartis, Rueil-Malmaison, France) administered daily, in combination with 2.5 mg/kg azathioprine administered on alternate days (Imurel; Glaxo-Welcome, Paris, France). A maximum of 10 mg/kg of ketoconazole (Nizoral; Janseen-Cilag, Issy-les-Moulineaux, France) was also added daily to decrease cyclosporine catabolism. Blood levels of cyclosporine were checked twice a week and cyclosporine doses were consecutively adjusted to maintain an immunosuppressive level between 250 and 350 ng/mL. The immunosuppressive regimen was started 1 week before the first cell administration, at an age ranging from 14 to 16 weeks, and maintained until the dogs were euthanized.

### Systemic cell delivery procedure

MuStem cell suspensions were prepared with a density range of 12 to 18x10^6^ cells/mL in 0.9% NaCl / 2.5% homologous serum / 10 U/mL heparin. Three injections of 6.5 to 23x10^7^ MuStem cells/kg, corresponding to two bilateral intra-arterial femoral injections and one injection into the cardiac left ventricle, were performed on 4 GRMD dogs (referred to as GRMD^MuStem^: 7G^Mu^ to 10G^Mu^) aged initially from 15 to 17 weeks, using laminar flow at a rate of 15 mL/min, as previously described [29]. To carry out the intra-cardiac injection, a 5-French angiographic catheter (100 cm long; Launcher coronary guide catheter, Medtronic, Tolochenaz, Switzerland) was advanced in a retrograde manner through the right carotid artery to the left ventricle. The effective crossing of the aortic valve with the catheter was assessed by appearance of a typical ventricular pressure curve.

### Clinical follow-up

Dogs were weighed and clinically assessed in a non-blinded manner by a D.V.M observer on a weekly basis during all the experimental protocol. Blood samples were collected monthly and hematological/serum biochemical testing were performed (S1 Fig). The clinical status of mock GRMD and GRMD^MuStem^ dogs was assessed weekly. A clinical score was measured following a previously described method [29,32]. Briefly, the clinical score was established based on 11 locomotion and muscular criteria and 6 items related to the general health status. It was expressed as the percentage of the maximum score defined as 100% for a healthy dog. Repeated-Measures ANOVA were carried out from 10 to 39 weeks of age.

### Muscle sampling

*Biceps femoris* muscle necropsies (0.5 cm^3^ fragments) were collected surgically from the middle portion of the muscle in 9-month-old healthy (#H1 to #H3), mock GRMD (#4G to #6G), and GRMD^MuStem^ (#7G^Mu^ to #9G^Mu^) dogs. Muscle biopsy was collected from the same muscle at 9 months of age from the #10G^Mu^ dog and used for histological analyses. This time point corresponds to four months after systemic administration of cells into GRMD^MuStem^ dogs. Muscle fragments were divided into two parts for histological and molecular analyses, and subsequently archived at -80°C until processing.

### Histology and immunohistochemistry

Transverse 8 μm-thick cryostat sections were stained with hematoxylin-eosin-saffron for histopathological assessment. Sections were incubated (overnight, 4°C) with primary antibody against dystrophin (1:50; NCL-DYS2, Novocastra) or the developmental isoform of myosin heavy chain (MyHCd, 1:100; Novocastra). 488 Alexa fluor conjugated goat anti-mouse IgG (1:300; Invitrogen) (1 h, room temperature (RT)) was used as a secondary antibody against dystrophin. Immunofluorescence labelling was observed with a laser scanning confocal microscope (Nikon C1; Champigny, France), and all acquisitions were performed as previously described [29]. For MyHCd, the sections were incubated with biotinylated goat anti-mouse (1:300; Dako) (1 h, RT) and streptavidin horseradish peroxidase (15 min, RT), which was revealed using DAB (3,3’-Diaminobenzidine) chromogen (10 min, RT). The sections were counterstained with a DRAQ5 dye (Biostatus, Leicestershire, UK) to stain the nuclei. Histomorphometric analyses, including measurements of fibre diameter and number of MyHCd positive fibres, were performed as previously described [29]. Mean fibre diameters were compared among different dog groups with analysis of variance followed by Fisher PLSD tests. Percentages of MyHCd^+^ fibres were compared between GRMD^MuStem^ and mock GRMD dogs using a Mann-Whitney test with a two-tailed P value. A value of P<0.05 was considered to be statistically significant. For dystrophin labelling, all acquisitions were performed with the same signal amplification resulting from identical detector gain value, as previously described [29]. To determine the proportion of dystrophin^+^ fibres, 1,121±167 total fibres were counted by using differential interference contrast in the *Biceps femoris m*uscle sections of the GRMD^MuStem^ dogs (n = 4) and sequentially the number of fibres expressing dystrophin was determined by DYS2 green fluorescent immunolabelling.

### RNA isolation

Total RNA was extracted from about 50 mg of frozen *Biceps femoris* muscle using the RNeasy Fibrous Tissue Mini Kit (Qiagen, Santa Clara, CA, USA) according to the manufacturer’s instructions. Total RNA was quantified using a NanoDrop spectrophotometer (Nanodrop Technologies, Wilmington, DE, USA). Each sample was made up by pooling two independent extractions of the same muscle and was then treated with DNAse Ambion (Life technologies, CA, USA). RNA quality was assessed using the Agilent Eukaryote Total RNA Nano kit with the 2100 Bioanalyzer (Agilent, Santa Clara, CA, USA).

### Canine gene expression microarray hybridization

The transcriptome experiment was performed by an Integrative Genomic Platform (Nantes, France). mRNA expression profiling was obtained using the Agilent-021193 Canine (V2) Gene Expression Microarray (Grid name: 021193_D_F_20120509- Protocol name: GE2_107_Sep09). Microarray hybridizations were performed using standard operating procedures and quality controls (Protocol: G4140-90050_GeneExpression_ Two_Color_v6.5-mai 2010). The labelling and staining were performed according to the Agilent protocol. Briefly, 100 ng of RNA were amplified and stained with cyanine -5 or cyanine-3 dyes (4 samples were stained with cyanine cy5 and 4 others with cy3). Pairs of differentially labelled samples (2x825 ng) were hybridized on a slide in 100 μL of hybridization buffer. After washing, the slides were scanned by the Agilent Scanner (Agilent Technologies Scanner G2505C). Agilent Feature Extraction software (V 10.7.1.1) was used to analyse the acquired array images.

### Raw data preprocessing

The microarray expression data were pre-processed and analysed using the AMEN (Annotation, Mapping, Expression and Network) suite of tools [33]. The data quality was checked against QC reports from Feature Number Version 10.7.1.1. A background correction was performed on the array data using the “normexp” method [34]. The microarray data quality was verified by plotting the log_2_ signal distribution across samples. The median expression values are considered for redundant probes. The array data of replicates were then normalized using the “quantile-quantile” method. Replicate intensity values of healthy, mock GRMD and GRMD^MuStem^ samples were averaged.

### Expression data analysis

Statistical filtration and classification were performed for each pairwise comparison of the samples: mock GRMD *versus* healthy dogs, GRMD^MuStem^ *versus* healthy dogs and GRMD^MuStem^ *versus* mock GRMD dogs. The detectable probes (22783/22841/22457) yielding signals ≥7.32 (median of the normalized expression dataset) were first identified in each of the three respective comparisons. A total of 1074 (605/940/99) probes were further selected displaying a high expression variation (fold-change, FC ≥2.0). A LIMMA statistical test [35] was then performed to identify 608 (430/567/41) probes that were significantly differentially expressed (F-value adjusted with the False Discovery Rate, p ≤0.05). For each of the three comparisons, the selected probes were finally classified into two groups (based on the fold-change), named “up” or “down” according to their expression profiles.

### Canine gene and probe annotation

To obtain the most complete annotation possible, we considered the dog annotation based on the Entrez Gene IDs (NCBI) provided in the AMEN suite of tools [33], and then supplemented with the Ensembl annotation. The microarray probes were subsequently mapped on the canFam3 genome using the BLAT program [36]. To filter the alignments, we considered a percentage of identity of ≥90% over the whole size of the probes (60 bps). The probes were further associated with canine genes based on their genome coordinates and the overlap with exonic regions of annotated loci. It is noteworthy that a given probe can be assigned to several genes, while several probes can be associated with a single gene.

### Gene Ontology enrichment analysis

The enrichments of annotation terms within a group of genes were calculated with the Fisher exact probability using a Gaussian Hypergeometric test. A Gene Ontology (GO) term is considered to be significantly enriched when the number of genes bearing this annotation is ≥3 and when the associated FDR-corrected p-value is ≤0.005 for biological process terms and ≤0.01 for terms related to the biological function and cellular component categories.

### Microarray data repository

Raw data CEL files are available via the EBI’s Array Express database (www.ebi.ac.uk/arrayexpress) under accession number E-MTAB-2095 [37].

### Comparison with transcriptomic studies of individuals with DMD

The group of genes significantly deregulated in muscle of individuals with DMD was extracted from four studies [8,9,11,12]. The conversion of the Affymetrix human probe set identifiers into dog NCBI Entrez Gene identifiers was performed with the AMEN suite of tools via the HomoloGene database [38] and the array annotations provided by the AILUN annotation platform [39]. A group of human genes is taken as significantly associated with a group of dog genes if the associated p-value is ≤0.005 (Hypergeometric test).

### Quantitative RT-PCR for validating Agilent dataset

The RT-qPCR validations were done on the three healthy dogs (#1H to #3H), on two mock GRMD dogs (#4G and #5G), on two GRMD^MuStem^ dogs (7G^Mu^ and 8G^Mu^). Additional RT-qPCR experiments were also performed on muscle total RNA from two non-immunosuppressed GRMD dogs (referred to as GRMD^nonIS^) kindly provided by collaborators.

A subset of genes was selected to validate the data obtained by microarray analysis. Reverse transcription reactions were carried out on 0.5 μg of total RNA using the GoScript reverse transcriptase (Promega, Madison, USA) in a total volume of 20 μL. All cDNA amplifications were performed, in duplicate or triplicate, using 1/20^th^ of the reverse transcription products and the MESA BLUE qPCR kit (Eurogentec, Seraing, Belgium). Quantitative PCR was run on the Thermocycler CFX96 (Biorad, California, USA) with the following parameters: 5 min at 95°C for the initial denaturation step, then 15 sec at 95°C, 1 min at 60°C per cycle, for a total of 40 cycles. The specific amplification was checked using a melting curve. Gene-specific oligonucleotide primers were designed using Oligo Primer Analysis Software v.7 (Molecular Biology Insights Inc., Cascade, USA) and synthesized by Eurofins MWG Operon (Ebersberg, Germany). The primers used are listed in Table 2. RPS18 was selected as an endogenous control. The relative expression levels were calculated by the 2^-ΔΔ Ct^ method.

**Table 2:** Sense and anti-sense primer sequences used for validation of quantitative real-time PCR expression.

Gene name	Transcript accession	Sense primer (5’-3’) sequence	Anti-sense primer (5’-3’) sequence	Product size (bp)
ACTC1	XM_535424.4	GGTGGGAATGGGACAGAAGG	TCACGGTTGGCCTTAGGGTT	219
ADIPOQ	NM_001006644.1	GAGATGGCACCCCTGGAGA	CCCCACACTGAATGC CGAAC	182
DEPTOR	XM_539149.4	CAGACCGGGGCATCATTCA	TTGACCCCTTCCTCTTCCCT	202
FBXO32	XM_532324.4	GACAAAGGACAGCTGGATTGGA	TCTCCGTACTGCTCTTTCCGTG	77
FLRT2	XM_005623720.1	GAGCTGCGAGTGGACGAAAA	AGGTTTGAGAAGGCCGTCAG	275
GATM	XM_544663.4	AAGTGATAGTGGGCAGAGCAG	GGGAAAATAGTGGCCTCCGTG	119
GPD1	XM_845287.2	AAGGACCAGACCCAAGGAC	AAGGCCCCACAGATCTCCA	104
HFE2	XM_854431.2	TGCTGGGGTTCCTCTTTCCT	CTTCCCTGTCTCTAACCCCT	139
MUSTN1	XM_005642250.1	TCGTACAGCACCCACCATGTC	TTCACACTCCCGCATGACC	160
NRBF2	XM_005618923.1	GGAAAAGGGCAAAGCGGGA	TGGAAGGGCTGTATTTCTCGG	147
PPP1R3B	XM_539996.3	TCGGTGTTCCTACGGTCTGTTC	CTTCCATCTCCACCTGCCT	135
PVALB	XM_003431505.1	CTTTACCGCTGTCGACTCCTT	TCTTGCCATCCCCGTCCTT	239
RPS18	NM_001048082.1	CTAGTGATCCCTGAGAAGTTCC	ATGTCTGCTTTCCTCAATACC	143
SPP1	XM_003434023.2	CCAGCAACCCAATTATTCACTCC	GGGAAAGTAGGACGGCATTGAAG	205
ST3GAL5	XM_005630478.1	AGCACAGGTACAGCACGGA	TCTCAAGTGTTCGGGCATGT	164
ZFAND5	XM_005615855.1	AGAGGCAGCAGAATAGTGG	AAACTGATGGACTGGGCTG	279

## Results

### Clinical follow-up of GRMD dogs

**Fig 1:**
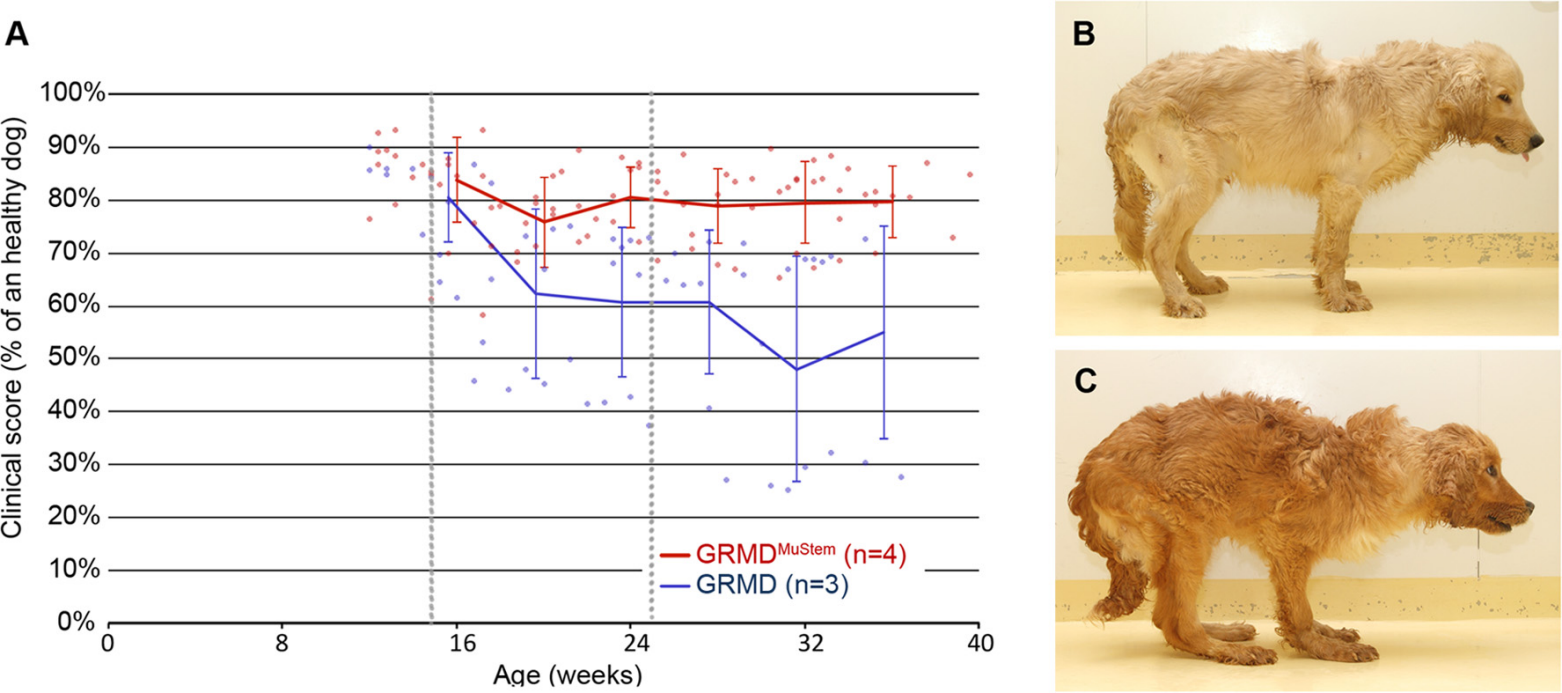
Clinical follow-up. (A) Mean±SD clinical scores of mock GRMD dogs and MuStem cell-injected dogs (GRMD^MuStem^). The clinical score of each GRMD dog was assessed weekly and expressed as a percentage of a theoretical healthy dog score. Limits of the MuStem cell delivery window are indicated (dashed lines). (B) Right lateral view of a GRMD^MuStem^ dog, #7G^Mu^. (C) Right lateral view of mock GRMD dog, #4G. Note the anterior weight transfer and plantigrady.

Our previous results demonstrated the capacity of wild-type MuStem cells to reach muscle tissue and generate clinical benefit following repeated injections into the left femoral artery of GRMD dogs [29]. Here, we reproduce the cell delivery protocol using a 6.5- to 23-fold increase in cell number and performing two bilateral intra-arterial femoral injections combined with an injection into the cardiac left ventricle of four immunosuppressed GRMD dogs. Mock dogs display a progressive clinical impairment as assessed by their clinical score (mock GRMD dogs; #4G to #6G) (Fig 1A). The clinical score is expressed as a percentage of the maximum score defined as 100% for a healthy dog. After a dramatic decrease of their ambulatory abilities from 14 to 20 weeks of age, their clinical score stabilizes at 56.7±21.2% at 38 weeks. This standard deviation illustrates the great variability in their clinical course as previously described [40]. All GRMD dogs that received MuStem cells (GRMD^MuStem^ dogs; #7G^Mu^ to #10G^Mu^) display an early and persistent stabilization of their clinical score that was maintained at 77.8±5.4% at 38 weeks (9 months of age) (Fig 1A). Repeated-Mesures ANOVA carried out from 10 to 38 weeks of age indicate a significant effect of the MuStem cell delivery (F = 601.5; p<0.001) and the individual GRMD dog (F = 143.9; p<0.001), as well as an interaction between cell delivery and time (F = 3.7; p<0.001). General posture and stance are the most obvious corrected criteria in GRMD^MuStem^ dogs (Fig 1B and 1C representing #7G^Mu^ and #4G, respectively). Overall, these data demonstrate that combined intra-femoral injection and cardiac ventricle deposit allow global and persistent improvement of the GRMD dog clinical status.

### Tissular impact of MuStem cell delivery

**Fig 2:**
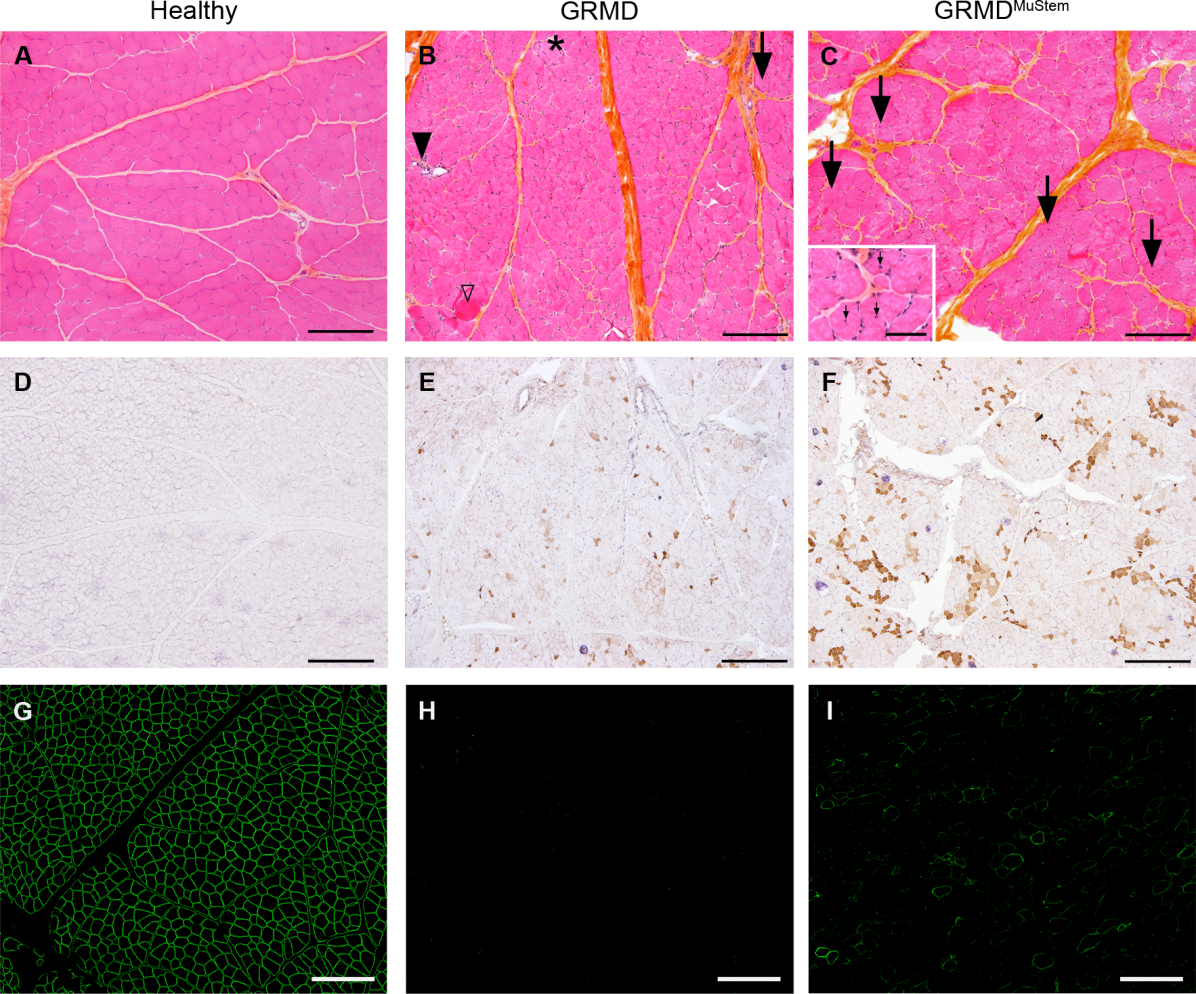
Histological analysis of the *Biceps femoris* muscle of 9-month old dogs. Healthy (#3H), GRMD (#6G), and GRMD^MuStem^ (#7G^Mu^) dog muscles are presented respectively in left, mid and right panel. (A, B, C) Muscle tissue presentation after hemalun eosin safran staining. (B) Mock GRMD dogs display a typical dystrophinopathic pattern with diffuse anisocytosis, hypertrophic hyaline fibres (open arrowhead), centronucleated fibres (arrow), necrotic fibres (*), regenerative foci (black arrowhead) and multifocal thickening of the endomysial space by fibrosis (highlighted by the saffron yellow staining). (C) The dystrophinopathic pattern is remodelled in GRMD^MuStem^ dogs: anisocytosis is milder, hypertrophic hyaline fibres and necrotic fibres are less numerous, whereas centronucleated fibres are more abundant (arrow) compared to GRMD dogs. Inset, another picture taken on the same tissue sample with a higher magnification that displays centronucleated fibre. (D, E, F) Regenerative activity of muscle fibres in healthy, GRMD and GRMD^MuStem^ dogs, as indicated after immunolabelling specific to the developmental isoform of the myosin heavy chain (MyHCd). (G, H, I) Dystrophin expression in healthy, GRMD and GRMD^MuStem^ dogs. (A-B-C; G-H-I) Scale bar = 200 μm (in set Scale bar = 100 μm). (D, E, F) Scale bar = 500 μm.

Histopathological analyses of skeletal muscles from all GRMD dogs show a typical dystrophic pattern including size variation among individual muscle fibres, individual fibre necrosis and calcification foci, as well as small regenerating basophilic fibres, numerous fibres with centrally located myonuclei and significant replacement of muscle tissue by connective tissue and, to a minor extent, adipose tissue (Fig 2A and 2B). Histomorphometry analysis was performed on the *Biceps femoris* muscle of 9-months aged healthy dogs (#1H to #3H), mock GRMD dogs (#4G to #6G) and compared to GRMD^MuStem^ dogs (#7G^Mu^ to #10G^Mu^). Based on the minimum Ferret diameter, we show that the mean fibre diameter is not discriminant between the groups (42.4±13.8, 34.8±16.8 and 33.0±14.2 μm for healthy, GRMD and GRMD^MuStem^ dogs, respectively). The variation coefficients associated with fibre diameter measurements (32.4, 48.3 and 42.9%, respectively) highlight the decreased anisocytosis observed in GRMD^MuStem^ dogs compared to mock GRMD dogs (p<0.0001). Regenerative activity in the same samples is also assessed using a specific MyHCd labelling (Fig 2D–2F). No MyHCd^+^ fibre are observed in healthy dog muscles. While 4.8±2.4% of fibres express this developmental isoform in mock GRMD dog muscles, the proportion of MyHCd^+^ fibre in GRMD^MuStem^ dogs is 18.7±9.5%, demonstrating an increase in muscle regenerative activity following MuStem cell delivery (p<0.001). Immunofluorescent labelling of dystrophin in the *Biceps femoris* muscle shows no expression of this protein in mock GRMD dogs, apart from some rare positive fibres corresponding to revertant fibres. On the other hand, isolated or clustered dystrophin^+^ fibres are scattered throughout the whole muscle section of GRMD^MuStem^ dogs. These fibres, corresponding to 25.0±3.9% of all fibres, are characterized by a global low expression level compared to that observed in healthy dog muscle and by a continuous or discontinuous labelling along the membrane (Fig 2G–2I and S2 Fig). A nuclear counterstaining and bright field-image allow us to visualize all the fibres in the muscle cryosection (S3 Fig). Altogether, we demonstrate histological remodelling of the GRMD dog muscle following MuStem cell delivery, including a boost in its regenerative capacity, which is consistent with the clinical impairment observed in the GRMD^MuStem^ dogs and a low-level expression of dystrophin in scattered fibres.

### Sample quality controls

The quality of total RNA samples was checked on the Agilent 2100 bioanalyzer. The electropherogram of the six samples corresponding to two healthy dogs (#1H, #2H), two mock GRMD dogs (#4G, #5G), and two GRMD^MuStem^ dogs (#7G^Mu^, #8G^Mu^), shows an absence of degradation products in parallel with high RNA Integrity Numbers (RIN), validating the quality of all initial RNAs (S4A Fig). The canine gene expression microarray data reveals a homogenous signal intensity distribution across the samples (S4B Fig). The dendrogram plotted from the distance matrix of the overall expression signals shows that the healthy samples are grouped together, revealing the proximity between the mock GRMD and GRMD^MuStem^ dog samples (S4C Fig).

### High-throughput characterization of GRMD dog model

**Fig 3:**
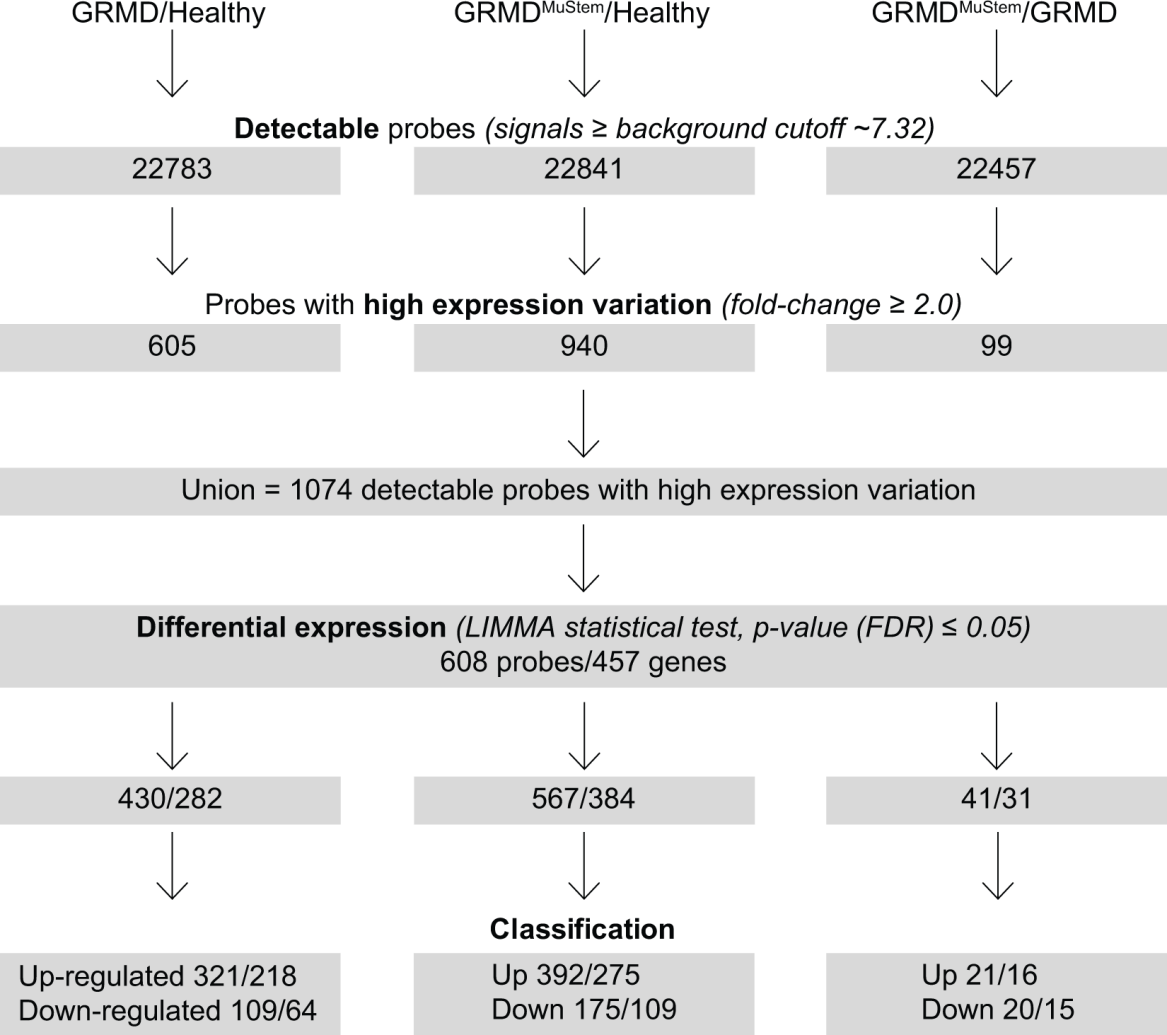
Flow chart of the statistical filtration of microarray data. Procedure leading to classification for each of the three pairwise comparisons of healthy, GRMD and GRMD^MuStem^ dog muscle samples.

The statistical filtration and classification (Fig 3) allows the identification of 282 genes (corresponding to 430 probes) differentially expressed between healthy and GRMD dogs, thus providing a high-throughput characterization of the GRMD dog model. Thus, 218 genes (321 probes) and 64 genes (109 probes) are identified as significantly up-regulated and down-regulated, respectively (S1 File.).

A comparison with human studies shows that, among the 282 genes differentially expressed between the GRMD and the healthy samples, 117 genes have been previously identified as differentially expressed in individuals with DMD in at least one of the studies considered [8,9,11,12]. Interestingly, out of these 117 genes, 98% are either up- or down-regulated in both the GRMD dog model and the DMD patient. As reported in Table 3, among the genes that are common between our dataset and the human transcriptomic studies, we can identify significant enrichments of genes that are up-regulated or down-regulated in both canine and human studies.

**Table 3:** Comparison with human studies.

		GRMD *versus* Healthy	
		Up-regulated	Down-regulated
Chen *et al*., 2000	Up-regulated	51 (2.9x10^-20^)	1
	Down-regulated	1	8 (4.9x10^-3^)
Haslett *et al*., 2002	Up-regulated	27 (3.6x10^-21^)	0
	Down-regulated	0	3 (3.9x10^-4^)
Haslett *et al*., 2003	Up-regulated	14 (9.4x10^-7^)	0
	Down-regulated	0	0
Pescatori *et al*., 2007	Up-regulated	76 (2.5x10^-44^)	0
	Down-regulated	0	15 (2.3x10^-11^)

The number of genes that are significantly up- or down-regulated in this dog model and in the human study are indicated. These numbers are followed by the corresponding p-value given in parentheses when a significant enrichment is detected.

**Fig 4:**
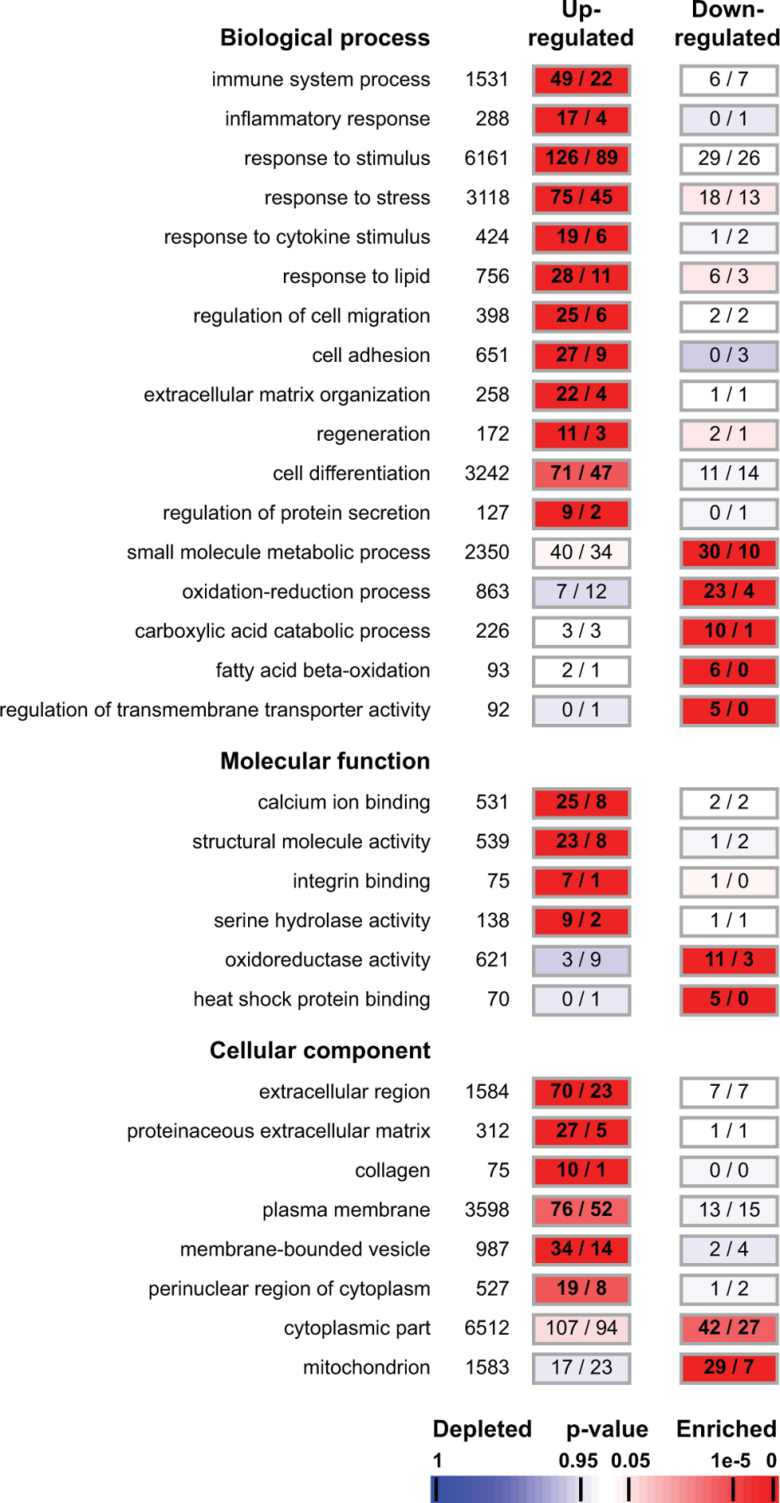
Functional characterization of GRMD dog model. A selection of Gene Ontology (GO) terms significantly enriched in the groups of genes up- and down-regulated between GRMD dog model and healthy dog. Associated with the GO term, the total numbers of genes assigned with this annotation are indicated, followed in the rectangle by the number of genes observed (on left) and expected by chance (on right). Numbers in bold indicate a significant over-representation of the corresponding GO term. Enrichments are colour-coded according to the associated p-value from red (enriched) to blue (depleted).

The GO terms significantly enriched in the group of genes differentially expressed between GRMD dog and healthy dog muscle are determined [41] (Fig 4 and S2 File). Among the genes up-regulated in GRMD dog as compared to healthy dog, we find enrichment for processes such as *immune system process* (n = 49, p<2x10^-5^), *inflammatory response* (n = 17, p<2x10^-4^), *response to stimulus* (n = 126, p<7x10^-6^), *response to stress* (n = 75, p<2x10^-4^), *response to lipid* (n = 28, p<4x10^-4^), *regeneration* (n = 11, p<2x10^-3^) and *cell adhesion* (n = 27, p<2x10^-4^). Calcium and energy metabolism as well as extracellular matrix disorders are also represented by the enrichment of *calcium ion binding* (n = 25, p<5x10^-5^) and *extracellular matrix organization* (n = 22, p<7x10^-8^) in the up-regulated group of genes. The down-regulated group is significantly enriched in genes associated with terms involved in *oxidation-reduction processes* (n = 23, p<6x10^-10^), *regulation of transmembrane transporter activity* (n = 5, p<5x10^-3^), *mitochondrion* (n = 29, p<7x10^-11^) and *fatty acid beta-oxidation* (n = 6, p<4x10^-4^). Furthermore, five genes involved in apoptosis regulation are noted among the group of genes which are up-regulated and involved in the *regulation of protein secretion* (n = 9, p<5x10^-3^). Also, we detect an enrichment of the *membrane-bounded vesicle* (n = 34, p<7x10^-5^) term in the group of up-regulated genes in GRMD dog muscle as compared to healthy dog.

### Impact of MuStem cell delivery on GRMD skeletal muscle

**Fig 5:**
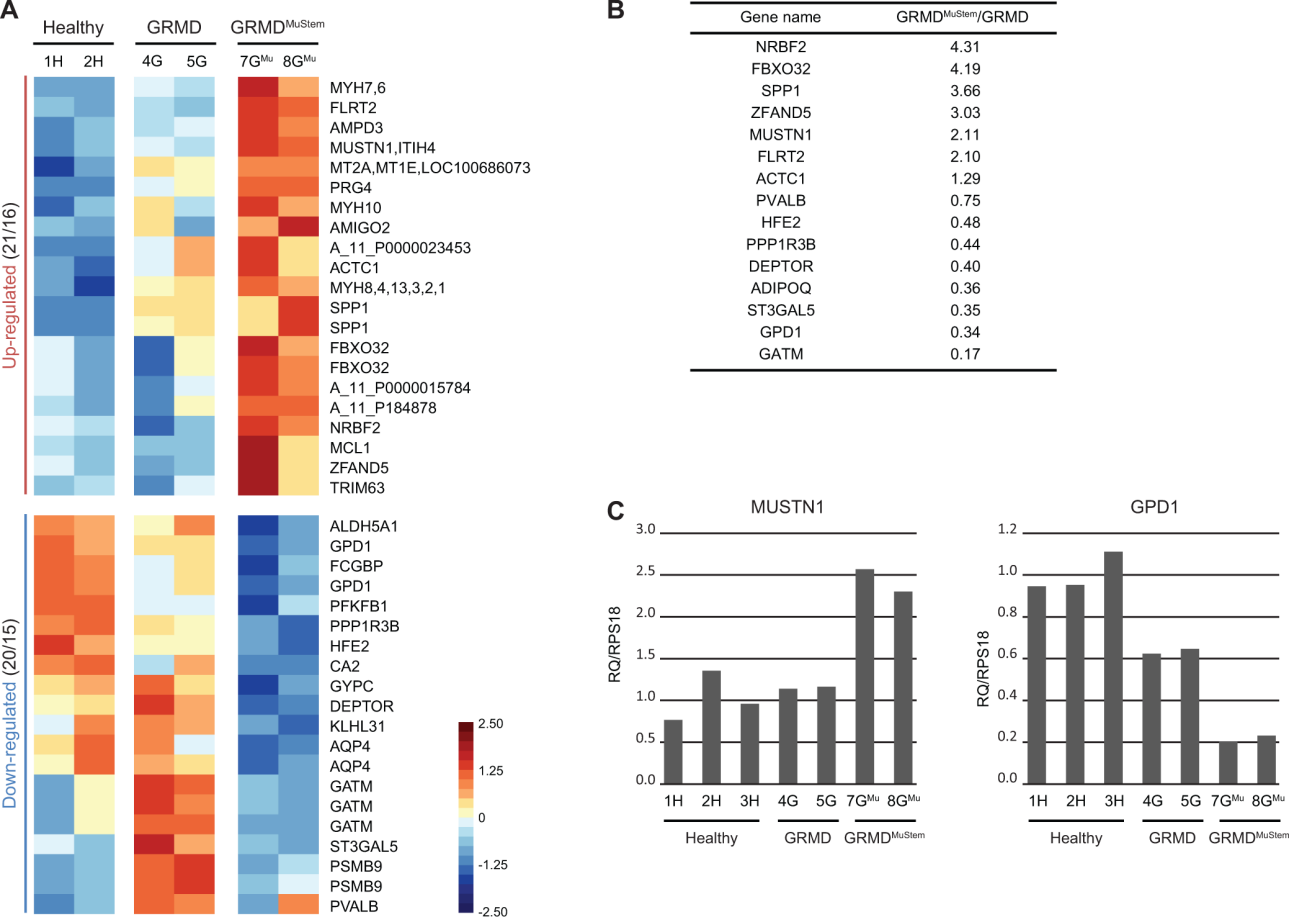
Impact of MuStem cell systemic delivery on GRMD dog muscle. (A) False-colour heatmap illustrating the transcripts significantly differentially expressed between GRMD^MuStem^ dog and mock GRMD dog samples. Each line and column corresponds to a probe and an individual biological sample, respectively. The gene names assigned to each probe are indicated on the right, separated by a comma in the case of multiple names as for example with the myosin isoforms. The number of probes / genes are indicated beside each expression profile name. The standardized log_2_-transformed intensities are plotted with a colour scale ranging from -2.50 (blue) to 2.50 (red) incremented by 0.25. (B) RT-qPCR validation of 15 genes. For each gene, fold change values are indicated for GRMD^MuStem^ dog *versus* GRMD dog. (C) Histograms of RT-qPCR for two selected genes: MUSTN1 and GPD1. Relative quantifications (RQ) are calculated by normalizing with respect to RPS18.

Thirty one genes (41 probes) are identified as differentially expressed as a consequence of MuStem cell administration (Fig 3). Sixteen genes (21 probes) are significantly up-regulated in GRMD^MuStem^ dog muscle as compared to mock GRMD dog. Their expression profile shows a global tendency to increase in mock GRMD dog samples as compared to healthy dog (Fig 5A). A significant down-regulation of expression is identified for 15 genes (20 probes) in GRMD^MuStem^ as compared to mock GRMD dog muscle. Among these 31 genes, 8 show a slight decrease of expression in GRMD samples as compared to healthy samples, followed by a marked decrease in GRMD^MuStem^ samples, while some other genes such as ST3GAL5, GATM or PSMB9 exhibit an increased expression in GRMD samples as compared to healthy sample followed by a significant decrease in GRMD^MuStem^ samples.

The number of genes in these expression patterns is too small for a GO statistical analysis. Nevertheless, it is noteworthy that we can identify genes involved in processes such as *muscle regeneration*, *cellular homeostasis* as well as *metabolism*. Thus, the activation of satellite cells (MUSTN1) and muscle regeneration (SPP1, MYH3/MYH8, DEPTOR, KLHL31 and PSMB9) are affected by MuStem cell delivery, in parallel with genes involved in protein degradation, protein folding (FBXO32, TRIM63, ZFAND5 and MT2A) and the anti-apoptosis pathway (MCL1, AMIGO2). Two genes (PVALB and AQP4) are implicated in cell homeostasis and calcium / water homeostasis, respectively. In terms of metabolism, two genes (ST3GAL5, GPD1) related to lipid metabolism are down-regulated. ADIPOQ represents a third example of the down-regulation of lipid metabolism. This latter gene is significantly up-regulated in GRMD dog muscle as compared to healthy dog, and has a tendency to be down-regulated in GRMD^MuStem^ dog (FC -1.57). Five other genes play a role in glucose metabolism (AMPD3, DEPTOR, GATM, PFKFB1 and PPP1R3B).

### Real-time quantitative PCR validation of array results

To validate the related microarray data, we chose 15 of the 31 differentially expressed genes in the GRMD^MuStem^ dog group compared to mock GRMD dog (Fig 5A) to carry out real-time quantitative RT-PCR analyses. The RT-qPCR validations were performed on the same sample set as the microarray (#1H, #2H, #4G, #5G, #7G^Mu^, #8G^Mu^) and also on the third healthy dog (#3H). To take account of the immunosuppressive context, we performed additional RT-qPCR experiments on muscle total RNA from two GRMD^nonIS^. Relative quantifications are calculated for healthy, GRMD^nonIS^ and mock GRMD dogs (S1 Table). GRMD dogs, whether or not immunosuppressed, show a similar variation of expression of the selected genes compared to healthy dogs. This reinforces the microarray data on the GRMD dog model.

Then, to validate the molecular impact of MuStem cell transplantation, FC values are calculated GRMD^MuStem^ *versus* GRMD dog comparison (Fig 5B). All of the genes further analysed in this way show expression changes consistent with those estimated by gene chip analysis. Interestingly, the two samples from the same group yield closely similar relative quantifications (RQ) for a given gene, as illustrated for the musculoskeletal embryonic nuclear protein 1 (MUSTN1) and the glycerol-3-phosphate dehydrogenase 1 (GPD1) genes (Fig 5C). In general, RT-qPCR nevertheless yields slightly higher values than those obtained in the microarray study, so this approach proves to be successful in validating microarray data.

## Discussion

Over the last fifteen years, myoblasts and several stem cell populations including mesoangioblasts, pericytes, CD133^+^ cells, PICs cells, Aldh^+^ cells, mesenchymal stem cells and Muscle-Derived Stem Cells (MDSC), have been tested in different dystrophic or damaged muscles and defined as having favourable engraftment properties and myogenic regenerative potential [42–50]. Interestingly, some of these cells also exhibit an unexpected ability to migrate through vessels, leading us to consider a whole-body distribution using systemic delivery [51]. As GRMD dogs represent a large animal model having a pathogenesis very close to individuals with DMD, they are highly appropriate for preclinical studies aiming to identify a new therapeutic product. In 2011, using this clinically appropriate animal model, we showed that intra-arterial delivery of allogenic MuStem cells, a sub-type of MDSC, resulted in a limitation in the development of muscle damage and a long-term stabilization of the transplanted GRMD dog clinical status [29]. These data positioned this population as a promising candidate for cell-based therapeutic development dedicated to DMD. This persistent and generalized effect leads us to question the molecular impact of MuStem cells; hence, in this study, we make use of a MuStem cell transplantation programme to develop an expression profiling analysis, along with an investigation of the clinical and histological status. In parallel with the first high-throughput characterization of GRMD dog muscle, our study presents, without prior assumptions, the molecular effects underlying the effect of MuStem cell transplantation.

### Pathophysiology of GRMD dog is defined by complex transcriptional remodelling

Considering the state of the art in this field, we need to establish, at a transcriptomic level, a referenced set of deregulated genes involved in the pathophysiology of GRMD dog. We find that up-regulated genes are significantly associated with genes bearing *regeneration*, *regulation of protein secretion* as well as *cell migration*. Three other biological processes: *extracellular matrix organization*, *inflammatory response* and *cell adhesion*, which are known as being modulated in individuals with DMD [8,17], are significantly enriched in the identified differentially expressed genes. Furthermore, in accordance with the description of large caveolae observed in the plasmic membrane of myocytes in GRMD dog [25], we observe an enrichment of the *membrane-bounded vesicle* term when comparing our GRMD samples with healthy dog samples. In addition, down-regulated genes in GRMD dog muscle are over-represented in metabolic processes, specifically involving the *carboxylic acid catabolic process*, *regulation of transmembrane transporter activity*, *fatty acid beta-oxidation*, as well as *oxidation-reduction* and *small-molecule metabolic processes*. These data are very well correlated with those previously obtained in individuals with DMD showing a differential gene profiling related to mitochondrial activity [52]. Overall, we show an altered expression profile of 282 genes, including 117 genes previously identified as differentially expressed in individuals with DMD in at least one of the studies concerned [8,9,11,12]. It is to notice that a limitation of the results presented here is that we compare immunosuppressed GRMD dogs with healthy dogs that were not submitted to an immunosuppressive regimen. This means that in such a comparison we observe the effect of the physiopathology associated with the effect of the immunosuppression known to impact the GRMD dog phenotype [53,54]. Despite this, importantly, 98% of these 117 genes are similarly differentially expressed in both GRMD dogs and individuals with DMD, and we show a significant enrichment of up-regulated or down-regulated genes in both dog and human studies, confirming that our experimental design in GRMD dog is appropriate for the investigation of DMD disease. These findings validate our strategy and also reinforce the evidence for molecular modifications observed after MuStem cell transplantation. Finally, our transcriptomic results obtained the first expression profile of GRMD dogs with an advanced age of 9 months yield new informative data on the expression profile of dystrophic dog muscle, clearly illustrating that the consequences of the lack of dystrophin are multiple and associated with a profound tissue remodelling, as shown by the involvement of a large number of biological processes.

### MuStem cell administration enhances long-term skeletal muscle fibre regeneration

In our previous study, we demonstrated that one of the consequences of MuStem cell systemic delivery is histological remodelling, with a major increase in the muscle fibre regeneration activity observed several weeks after cell delivery. The skeletal muscle samples from the dogs used in the present study similarly show a striking increase in the proportion of newly formed/regenerated muscle fibre, which is revealed by the expression of developmental myosin heavy chain isoform in transplanted GRMD dog muscle four months after systemic administration. This result highlights that transplanted MuStem cells actively contribute to persistent stimulation of muscle fibre formation, as shown at the molecular level by the identification of up-regulated genes including MUSNT1 (musculoskeletal embryonic nuclear protein1) and SPP1 (Secreted PhosphoProtein 1), which are implicated in myogenesis, regeneration and cell differentiation. MUSNT1 is a key regulator of myogenic differentiation and fusion [55], and has been shown to be up-regulated following the differentiation of porcine myoblasts into myotubes [56]. Interestingly, the expression of this gene has been recently demonstrated in adult regenerating skeletal muscle, activated satellite cells and differentiating myoblasts [57]. SPP1, also named osteopontin, is an adhesive component of the extracellular matrix that also exists as a soluble molecule. It is described as a key cytokine regulating tissue repair, inflammation and fibrosis [58,59]. In injured muscle, SPP1 promotes macrophage binding to necrotic fibres, and thus may be important in mediating the early phase of muscle regeneration [60]. More recently, myoblasts have also been presented as an important source of SPP1 in damaged muscles, where its release may assist in controlling both myogenic and inflammatory processes during the early stages of muscle regeneration [61]. Concomitantly, we also find that developmentally regulated genes, known to be transiently expressed during muscle development and regeneration, such as ACTC1 (actin, alpha, cardiac muscle 1) and MYH3/MYH8 isoforms are significantly up-regulated in the GRMD^MuStem^ dog with advancing age. Interestingly, such enhanced expression of ACTC1, embryonic myosin heavy chain, versican, perinatal myosin heavy chain, embryonic myosin light chain has also been documented in individuals with DMD [9]. Lastly, we observe a decreased expression of the DEP domain containing MTOR-interacting protein (DEPTOR), which is described to negatively regulate muscle proliferation and differentiation via the regulation of cell cycle regulatory proteins as well as muscle protein synthesis [62]. The results of this transcriptome analysis collectively reveal for the first time a molecular signature confirming the contribution of MuStem cells to muscle fibres regeneration and repair.

### MuStem cell administration enhances protein degradation in an ubiquitin-dependent manner

The regulatory role of the ubiquitin proteasome system (UPS) ensures the clearance of dysfunctional or denatured proteins. In a muscular context, the UPS has been shown to have a role in the mediation of skeletal muscle atrophy [63–65]. In a pathological context such as myopathy, skeletal muscle requires a rapid and efficient system for the removal of altered organelles, the elimination of protein aggregates, and the disposal of toxic products that may lead to cell death, thus allowing for the proper contraction of sarcomeres [66]. The UPS is thus involved in protein quality control, and the two ubiquitin ligases atrogin-1 and MURF1, which are associated with autophagy-lysosome systems, can ensure the rapid elimination of single proteins or small aggregates [67–69]. Autophagy is required for cellular survival, and this new concept changes the current view that proteolysis is detrimental; the new vision is to consider transitory activation of the proteolytic systems to eliminate misfolded muscle proteins in myopathy [69]. In human heart failure syndromes, recent evidence supports a role for protein damage and impaired clearance of damaged proteins in the pathology, and highlights the UPS as the primary effector of regulatory control [70]. In the present study, we show that MuStem cell transplantation stimulates the expression of several genes involved in the protein degradation machinery via this system. Specifically, FBXO32 (Atrogin 1), TRIM63 (MURF1), ZFAND5 and MT2A encode proteins of the E3 ubiquitin ligase complex, which mediates the ubiquitination, and subsequent proteasomal degradation of target proteins. This suggests that muscle tissue remodelling resulting from MuStem cell transplantation may involve preliminary UPS proteins and associated proteins such as metallothioneins in order to clear misfolded proteins. These latter could be toxic to myogenic cells, and have to be eliminated prior to efficient muscle fibre formation. After MuStem cell transplantation, a reversal is observed in the expression level of the subunit LMP-2 (PSMB9 gene), which is significantly up-regulated in GRMD dog as compared to healthy dog, strongly suggesting that protein degradation required for skeletal muscle regeneration is mainly mediated in an ubiquitin-dependent manner.

### MuStem cell administration induces insulin resistance in skeletal muscle

Based on under-representation of several key enzymes that control both glycolytic and oxidative metabolism, we have previously shown a dramatic alteration of metabolic proteins in GRMD dog *versus* healthy dog [6]. Skeletal muscle is responsible for 70 to 80% of whole body uptake, disposal, and storage of insulin-stimulated glucose. Therefore, we consider that skeletal muscle plays a major role in energy balance. Furthermore, skeletal muscle has an influence on the metabolism and storage of lipids and plays an important role in hormone signalling [71]. Insulin resistance in adipocytes induced by TNFα is accompanied by an increased GM3 biosynthesis through the up-regulation of GM3 synthase (ST3GAL5) gene expression [72]. In the present study, we find that the level of GM3 synthase mRNA is significantly higher in GRMD dog muscle than in healthy dog, possibly reflecting adipose tissue infiltration and, more generally, the participation of GM3 synthesis in abnormal insulin resistance in GRMD dog. It is interesting to note that, after MuStem cell transplantation, skeletal muscle is characterized by a reversal in the expression level of ST3GAL5, which could represent the molecular hallmark of a decrease in infiltrating adipose tissue, triglyceride synthesis and an improved insulin signalling after cell transplantation [73]. This hypothesis is supported by the fact that, similarly, the expression of Glycerol-3-phosphate dehydrogenase 1 (GPD1), equally involved in insulin metabolism, is significantly decreased in GRMD^MuStem^.

Muscle glycogen accumulation results from the disruption of the insulin pathway influencing glucose metabolism and transport, and has been demonstrated in *mdx* mice [74] and individuals with DMD [75,76]. Such accumulation is accompanied by elevated glycogen synthase activity and protein expression. In a previous proteomic study performed on GRMD dog muscle, we observed an increased level of protein phosphatase 1 (PP1) concomitantly with a decrease in the level of its major deactivator, glycogen synthase kinase (GSK)-3β [26]. Consistent with this result, we demonstrate the down-expression in GRMD^MuStem^ dog muscle of both genes encoding the proteins PP1R3B (Protein Phosphatase 1, regulatory subunit 3B) and PVALB (parvalbumin) that are involved in glycogen synthesis. These genes belong to the same regulatory network as the muscular adiponectin (ADIPOQ), which is known to regulate glucose and fatty acid metabolism both directly and via insulin sensitizing effects [77]. Also, it is proposed that its up-regulation probably reflects adipose tissue infiltration [78]. Here, we observe a significant over-expression of this gene in GRMD dog muscle, followed by a slight tendency to decreased gene expression four months after MuStem cell transplantation, which is confirmed by RT-qPCR. Since adiponectin elicits important functional effects on skeletal muscle [79] and can generate beneficial metabolic effects, as recently established in diabetes [80], while its local production is also associated with increased insulin sensitivity [81], further experiments need to be performed to refine the role of MuStem cells in the adiponectin-dependent regulatory pathway. Overall, these new molecular results give compelling evidence suggesting that MuStem cells influence insulin resistance and glucose and fatty acid metabolism in GRMD dog muscle.

### MuStem cell administration causes a lower requirement of creatine synthesis

L-arginine:glycine amidinotransferase (GATM or AGAT) is a mitochondrial enzyme that belongs to the amidinotransferase family and catalyzes the first step of creatine synthesis. It is involved in many metabolic (threonine, serine, arginine, proline) pathways, in creatine biosynthesis and in tissue regeneration [82]. GATM was found to be up-regulated in *mdx* mice, and it has been proposed that the up-regulation of the creatine synthetic pathway may help maintaining muscle creatine levels and limiting cellular energy failure in *mdx* skeletal muscles [83]. Among the differentially expressed genes identified herein, GATM exhibits an up-expression in GRMD muscle that could directly point out depletion of the muscle creatine pool occurring in highly and continuously remodeling muscle tissue. Interestingly, a 2-fold decrease was determined four months after MuStem cell transplantation, based on independent analysis of three probes. Then, GATM expression level is similar to the one in the healthy dog muscle. This could evoke a lower requirement of creatine synthesis correlated to a better tissue organization following an increased regeneration of muscle fibre.

### Conclusions

In summary, this study shows for the first time the effect of an adult stem cell transplantation on skeletal muscle gene expression in a DMD-like context. Interestingly, our results clearly reflect that MuStem cells impact many biological processes several months after their transplantation that could explain the beneficial effect on dystrophic phenotype of GRMD^MuStem^ dogs. Among these effects, gene expression profiling demonstrated an ability to maintain an intense muscle fibre regeneration activity that may play an essential role in stabilization of muscle tissue phenotype.

## Supporting Information

S1 ARRIVE Checklist The ARRIVE Guidelines Checklist. Animal Research: Reporting In Vivo Experiments.
(PDF)

S1 Fig Weight curve and biochemical parameters of GRMD and GRMD^MuStem^ dogs. Dogs were weighed and underwent weekly veterinary examinations during treatment. Creatine kinase (CK), urea, creatinine, alkaline phosphatase (ALP) and alanine aminotransferase (ALT) were determined for GRMD dogs (#4G to #6G) and GRMD^MuStem^ dogs (#7G^Mu^ to #10G^Mu^). Dogs that received MuStem cell transplantation are shown in grey. *References values were obtained from Oniris (Nantes, France). ^#^Dogs are not yet under immunosuppression at this time point.
(TIFF)

S2 Fig Dystrophin expression profile in supplementary GRMD^MuStem^ dog muscle samples. Transverse cryosections of the *Biceps femoris* muscle of 9-month-old healthy (A) #2H, GRMD (B) #5G and additional GRMD^MuStem^ dogs (C-E; #9G^Mu^, #8G^Mu^ and #10G^Mu^ respectively). Scale bar = 200 μm.
(TIF)

S3 Fig Combined Dystrophin immunolabelling and nuclear counterstaining. The first panel is a bright field image (A-C). Dystrophin expression in healthy (D), mock GRMD (E) #5G and GRMD^MuStem^ (F) #9G^Mu^ dogs. Nuclear counterstaining is shown in red. Scale bar = 200 μm.
(TIFF)

S4 Fig Total RNA and microarray data quality controls. (A) Total RNA electropherograms. The RNA integrity numbers (RIN) are calculated for each sample. (B) Distribution of the log_2_-transformed signal intensities of the duplicate healthy, GRMD and GRMD^MuStem^ dog samples before normalization. (C) Distance matrix of normalized expression data. The similarity between samples are colour-coded from white for the identity to black for the most dissimilar, allowing a clustering of the samples according to the dendrogram represented at the top and on the left.
(EPS)

S1 File The file summarizes the 608 probes identified as differentially expressed in this study. Several types of information such as gene name, annotation and classification are available, as well as comparisons with other human studies. Normalized and log_2_-transformed expression data are given for individual dog samples and averaged for healthy, GRMD and GRMD^MuStem^ groups.
(XLSX)

S2 File The file contains the results of the Gene Ontology enrichment analysis for the group of genes differentially expressed in the GRMD sample as compared to the healthy dogs. “n” represents the total number of genes assigned using the Gene Ontology terms and in the considered group of genes. “nEXP” corresponds to the number of genes expected by chance.
(XLSX)

S1 Table RT-qPCR values of the 15 selected genes for healthy, non-immunosuppressed GRMD (GRMD^nonIS^) and GRMD dogs. (PDF)
