# Supplementary figures and images for: Differential Gene Expression Profiling of Dystrophic Dog Muscle after MuStem Cell Transplantation
Source: PLoS One. 2015 May 8;10(5):e0123336. doi: 10.1371/journal.pone.0123336 (PMC4425432; doi:10.1371/journal.pone.0123336)

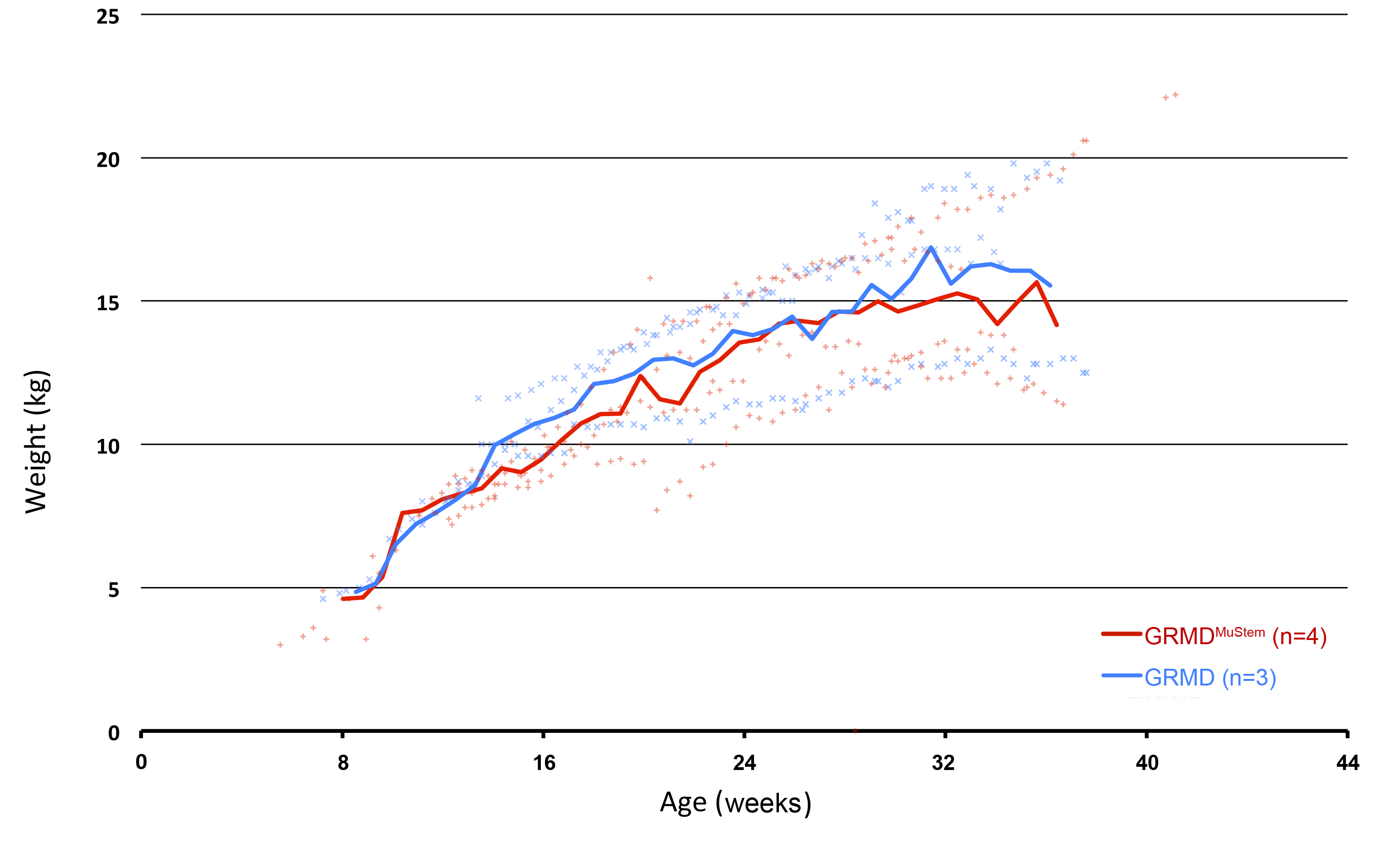

Supplement: S1 Fig — Dogs were weighed and underwent weekly veterinary examinations during treatment. Creatine kinase (CK), urea, creatinine, alkaline phosphatase (ALP) and alanine aminotransferase (ALT) were determined for GRMD dogs (#4G to #6G) and GRMDMuStem dogs (#7GMu to #10GMu). Dogs that received MuStem cell transplantation are shown in grey. *References values were obtained from Oniris (Nantes, France). #Dogs are not yet under immunosuppression at this time point. (TIFF) [file pone.0123336.s002.tiff]

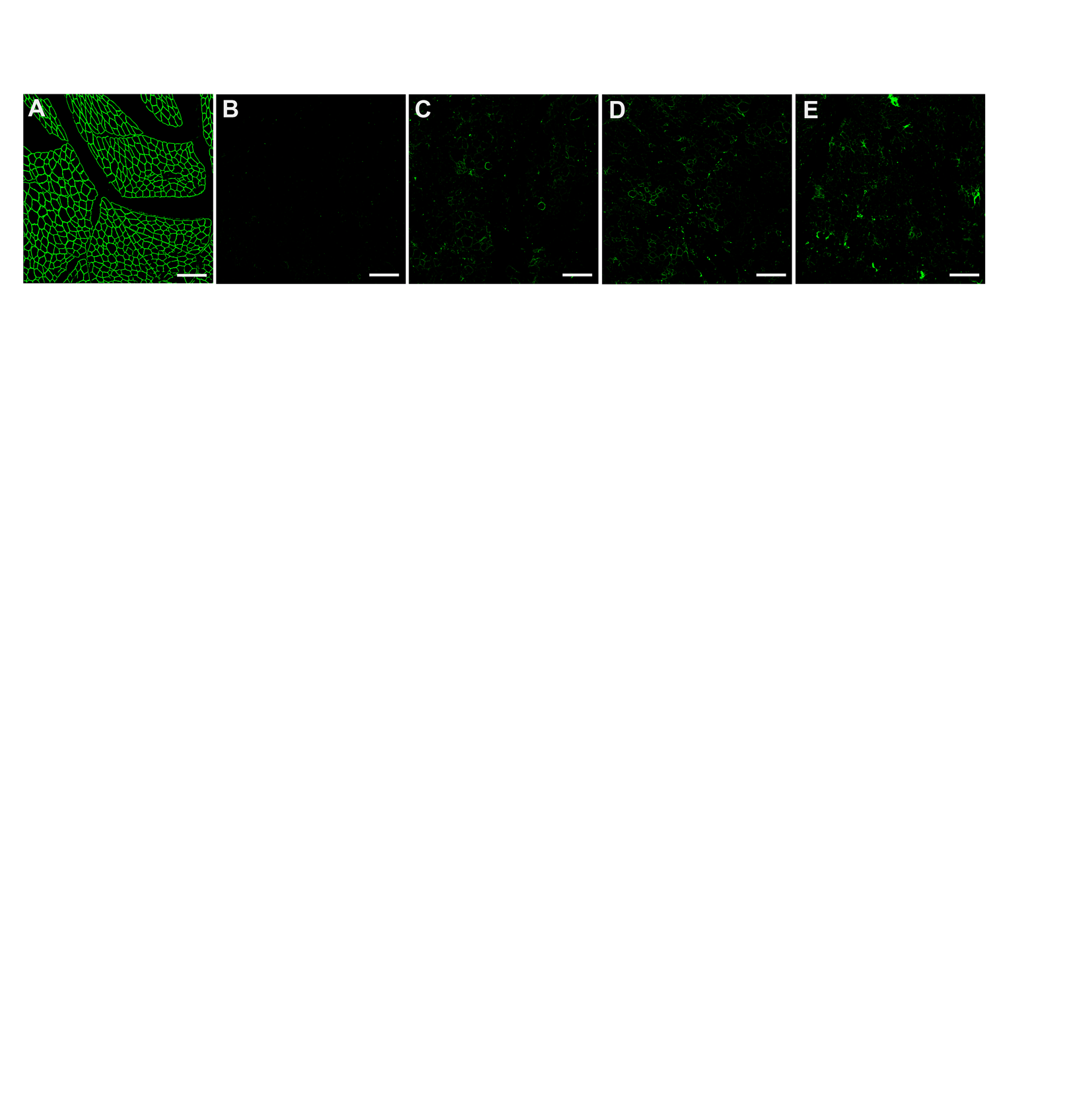

Supplement: S2 Fig — Transverse cryosections of the Biceps femoris muscle of 9-month-old healthy (A) #2H, GRMD (B) #5G and additional GRMDMuStem dogs (C-E; #9GMu, #8GMu and #10GMu respectively). Scale bar = 200 μm. (TIF) [file pone.0123336.s003.tif]

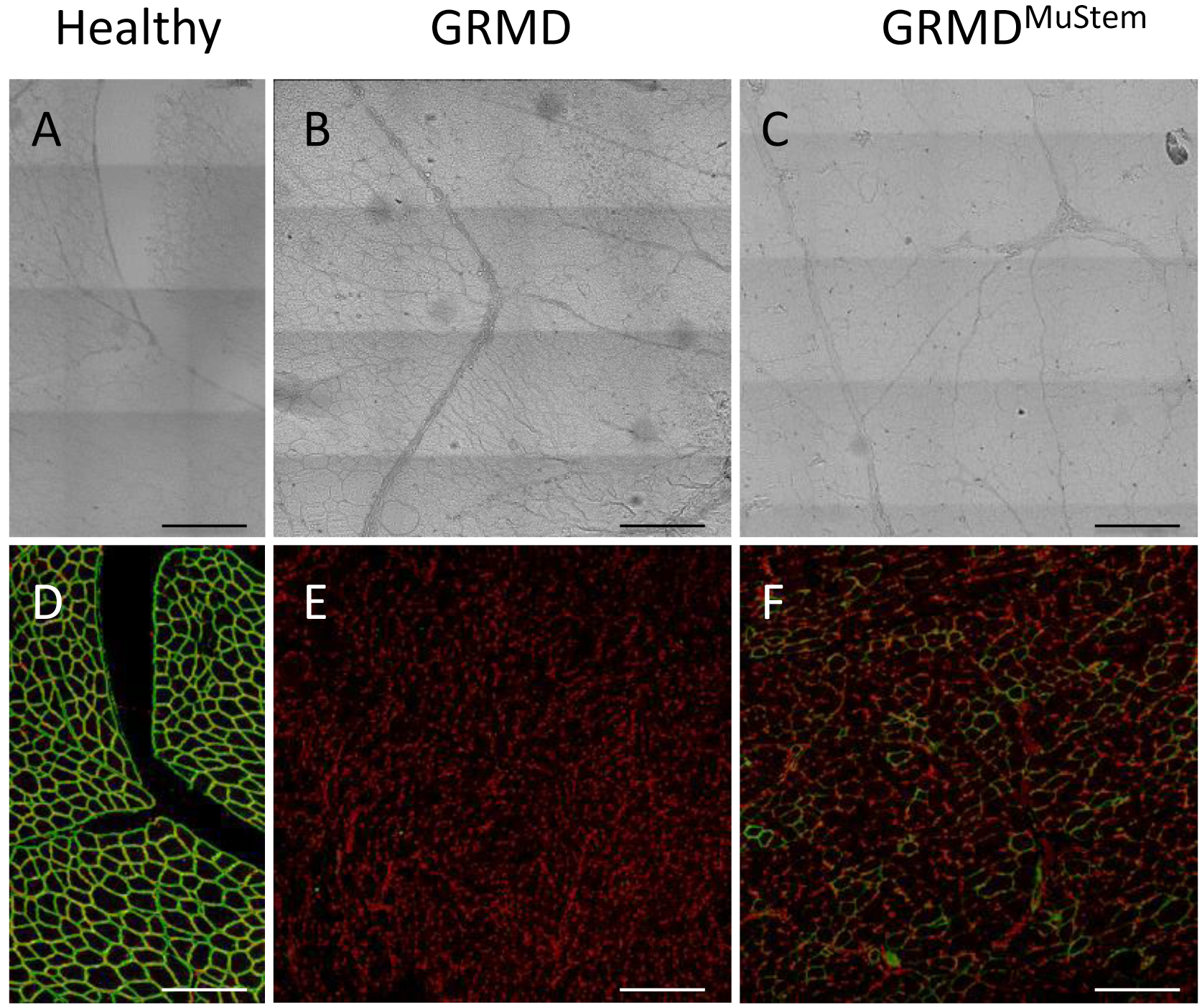

Supplement: S3 Fig — The first panel is a bright field image (A-C). Dystrophin expression in healthy (D), mock GRMD (E) #5G and GRMDMuStem (F) #9GMu dogs. Nuclear counterstaining is shown in red. Scale bar = 200 μm. (TIFF) [file pone.0123336.s004.tiff]
