# Supplementary material for: Differential Gene Expression Profiling of Dystrophic Dog Muscle after MuStem Cell Transplantation
Source: PLoS One. 2015 May 8;10(5):e0123336. doi: 10.1371/journal.pone.0123336 (PMC4425432; doi:10.1371/journal.pone.0123336)
Supplement: S1 Table — (PDF) [file pone.0123336.s008.pdf]

	Healthy	GRMD^{nonIS}	GRMD
**NRBF2**	1.02	0.64	0.77
**FBXO32**	1.03	1.10	1.11
**SPP1**	1.12	13330.69	270.15
**ZFAND5**	1.01	1.33	0.65
**MUSTN1**	1.03	1.62	1.15
**FLRT2**	1.11	0.26	0.85
**ACTC1**	1.28	1.96	1.66
**PVALB**	1.07	21.62	24.42
**HFE2**	1.00	0.36	0.55
**PPP1R3B**	1.02	0.26	0.50
**DEPTOR**	1.00	1.10	1.18
**ADIPOQ**	2.12	45.74	39.49
**ST3GAL5**	1.01	3.37	1.71
**GPD1**	1.00	0.66	0.63
**GATM**	1.16	1.08	2.53
